# Biomechanics in Musculoskeletal Health

**DOI:** 10.1155/2017/8916431

**Published:** 2017-10-30

**Authors:** Wenxin Niu, Ming Zhang, Jie Yao, Liping Wang, Ka-Chun Siu

**Affiliations:** ^1^Shanghai Yangzhi Rehabilitation Hospital, Tongji University School of Medicine, Shanghai 201619, China; ^2^Department of Rehabilitation Sciences, Tongji University School of Medicine, Shanghai 200092, China; ^3^Interdisciplinary Division of Biomedical Engineering, The Hong Kong Polytechnic University, Hung Hom, Hong Kong; ^4^Key Laboratory for Biomechanics and Mechanobiology of Ministry of Education, School of Biological Science and Medical Engineering, Beihang University, Beijing 100191, China; ^5^Sansom Institute for Health Research and School of Pharmacy and Medical Sciences, University of South Australia, Adelaide, SA 5001, Australia; ^6^College of Allied Health Professions, University of Nebraska Medical Center, Omaha, NE 68198, USA

Musculoskeletal problems, such as traumatic injury, osteoporosis, and osteoarthritis, are one of the leading causes of disability worldwide and are therefore a crucial branch of public health. Biomechanics has been proven to play a critical role in musculoskeletal pathology, treatment, and rehabilitation. Biomechanical factors could influence cell's metabolic activity, bone remodeling, and sequelae development. Advancement in biomechanical theory, methodology, and technique could also promote the improvement on the design of protective, surgical, and rehabilitation equipment. Advancement in computational technique even promoted the development of computational biomechanics.

The focus of this special issue is on innovative theory and application of biomechanics to understand musculoskeletal pathology and to improve the techniques for the treatment and rehabilitation. This special issue can serve as a platform for biomechanical researchers, rehabilitation therapists, protective device designers, and orthopedic surgeons.

Finite element (FE) analysis is a powerful tool for biomechanical investigations, especially in orthopedic surgery. Two independent research groups from the School of Medicine, Tongji University, respectively, constructed two separate three-dimensional FE models of lumbar spine (L3-L5). Z. Zeng et al. studied the biomechanical effect of different grades of facetectomy, while M. Yang et al. evaluated the stability of extraforaminal lumbar interbody fusion and traditional transforaminal lumbar interbody fusion under various internal fixations.

Researchers from Tianjin University of Technology also conducted a study in FE modeling. They developed and validated a refined FE model of middle femoral comminuted fracture to compare the biomechanical stability after two kinds of plate fixations. H.-Y. Liu et al. established a solid-liquid coupling biphasic model of articular cartilage and a microscopic model of chondrocytes to analyze the biomechanical response under cyclic compressive loading. Additionally, they performed a comprehensive analysis about the impact of pectus excavatum on scoliosis and elaborated its biomechanical mechanism in pectus excavatum patients with scoliosis. Y. He et al. studied the biomechanical performance of the laparoscopic-assisted plate.

Mechanism and protection of sports injury are another focus of this special issue. A researcher from Shanghai University of Sports contributed two articles. R. Xia et al. determined the effects of fatigue on the impact forces and sagittal plane kinematics of the lower extremities of recreational athletes in a drop-landing task. They also studied joint torque and mechanical power of lower extremities and its relevance to hamstring strain during sprint running. A group of researchers from Shenyang Sport University determined the contact force loading associated with different walking speeds.

Biomechanics is also very popular in rehabilitation in recent years. X. Wu et al. studied early spatiotemporal patterns and knee kinematics during level walking in individuals following total knee arthroplasty (TKA). An article contributed by authors from Taiyuan University of Technology, University of Sussex, and University of Southampton studied the effects of overweight and obesity on TKA. Y.-P. Huang et al. investigated the effect of arch support insoles on uphill and downhill walking of persons with flatfoot. S. Kuai et al. examined the compensatory response of the muscle activities of seventeen major muscle groups in the spinal region, intradiscal forces of the five lumbar motion segment units.

This special issue also included cell biomechanics and other topics. To achieve the accurate strain control of the membrane during stretching, a strain feedback compensation method based on the digital image correlation is proposed by authors form Sichuan University. Y. Chen et al. investigated the effect of endogenous *n*-3 PUFAs on fracture healing by measuring femur fracture repair in both *fat-1* transgenic mice and WT mice. Y.-X. Zhang et al. investigated the effects of different extracts of propolis and component of flavonoids on platelet aggregation.

Overall, this special issue covers a wide range of biomechanics in musculoskeletal health. More research is needed to investigate further in computational modeling in bones, sport injury prevention, rehabilitation, and cellular level to advance the field of biomechanics.

*Wenxin Niu* *Ming Zhang* *Jie Yao* *Liping Wang* *Ka-Chun Siu*

